# Perception and level of satisfaction with the quality of antenatal care services among pregnant women attending antenatal clinic at plateau state specialist hospital, Jos, Nigeria

**DOI:** 10.4314/ahs.v23i3.7

**Published:** 2023-09

**Authors:** Olaoye Titilayo, Oyerinde Oyewole, Aguiyi Chidera, Mercy Omosuzi

**Affiliations:** 1 Babcock University, Public Health; 2 Babcock University, Social Works

**Keywords:** Antenatal care, perception, level of satisfaction, Plateau

## Abstract

**Objective:**

This study evaluated pregnant women's perception and level of satisfaction with quality of antenatal care received at Plateau State Specialist Hospital.

**Materials and Methods:**

The study employed a hospital-based cross-sectional design. Convenience sampling was used to select two hundred and thirty-eight (238) women attending antenatal clinic at the Plateau State Specialist Hospital. A validated semi-structured interviewer-administered questionnaire with a Cronbach's Alpha score of 0.83 was used for data collection. Respondent's perception was measured on a 36-point rating scale and the level of satisfaction on a 9-point rating scale. Data was subjected to descriptive analysis and Pearson's correlation was used to test for hypothesis.

**Results:**

The mean age was 28.79 years. Most of the respondents were married (81.9%), twenty-eight percent of the respondents had two children. The respondent's perception of quality of antenatal care shows a mean score of 25.0± 4.61 which indicates that the respondents had positive perception of the quality of antenatal care (69.4%). Also, the respondent's level of satisfaction with the quality of antenatal care received shows a mean score of 6.50 ± 1.8 indicating that they were very satisfied (72.2%) with quality of antenatal care. There was a significant relationship between the respondent's perception of the quality of antenatal care and level of satisfaction of the quality of antenatal care service (r= 0.43; p< 0.003).

**Conclusion:**

The respondents had positive perception and are satisfied with the quality of antenatal care services received. A Periodic patient satisfaction survey should be established as part of the antenatal evaluation to provide feedback for continuous quality improvement.

## Introduction

As entry point to maternal health care services, antenatal care (ANC) has a potential for reducing maternal and neonatal mortalities by detecting at risk pregnancy and managing the risk involved^1^. It provides a platform for important healthcare functions including health promotion, counselling, education, screening, diagnosis, and disease prevention^2^. The importance of ANC service is underscored by the third sustainable development goal with a target to reduce global maternal mortality rate to fewer than 70 maternal deaths per l00,000 lives birth 3. The maternal mortality ratio for Nigeria in 2018 was estimated to 451 deaths per 100,000 live births 4. In regions with the highest rates of maternal mortality, such as sub-Saharan Africa and South Asia, even fewer women received at least four antenatal visits (52% and 46% respectively) 5. The impression on the quality of services received has been identified as a barrier to the utilization of antenatal services and the levels of satisfaction of the antenatal care attendees also has a significant influence on the utilization of antenatal care services 6. Perception of care by pregnant women is an important aspect of quality antenatal care as it influences their assessment of the services provided and determines if the woman will register and continue with the service^5^. Patient satisfaction has been traditionally linked to the quality of services rendered and the extent to which specific needs are met. Satisfied patients have a high probability of returning for follow ups, take medications and also recommend the services to others 2.

Research has shown factors responsible for patient satisfaction includes attitude of staff, time spent at the hospital, availability of doctors and doctor-patient communication 7. A number of studies have identified the lack of antenatal care as a risk factor for maternal morbidity and mortality 8,9. Antenatal care service utilization in some parts of Nigeria is low due to poor quality services which may be associated with finance, accessibility and patient waiting time 6,10. To reduce maternal mortality and increase access to care in Nigeria, patient satisfaction is a valuable tool and continued use of the services is more likely if there is a high level of satisfaction 11. Since inadequate ANC is associated with worse pregnancy outcomes, it is vital for health policymakers to better understand the factors influencing appropriate and timely utilization of ANC. Utilization of services during pregnancy may lead to further utilization of additional maternal services like institutional delivery and seeking assistance for complications during delivery and postnatal period 12.

Several studies have been conducted on perception and satisfaction of antenatal care in some African and Asian countries 13, 14, 15 to including Nigeria. The few studies that have been conducted in Nigeria are geographically restricted neglecting the North central zone of the country 16, 17,18,19. There are paucity of study that addressed the perception and level of satisfaction of antenatal care in Plateau state, Nigeria. This study therefore examined the perception and level of satisfaction with the quality of antenatal care services among pregnant women at Plateau State Specialist Hospital.

## Materials and methods

### Study area and design

The research employed a hospital-based cross-sectional design. This study was conducted in Plateau State Specialist Hospital located in Angware town in Jos East Local Government Area of Plateau State, Nigeria. It has an area of 1,020 km^2^ and a population of 85,602 at the 2006 census. It is a tertiary Health institution that provides specialized medical services, trains health professionals and serves as a research center.

The administrative department, medical and radiology department, nursing department, pharmacy department, and laboratory department are the five major departments at Plateau State Specialist Hospital. There are two antenatal clinics per week, one booking on Wednesday and two postnatal clinics on Tuesday and Thursday respectively. The clinic usually commences with an interactive health talk coordinated by a qualified community health nurse which usually lasts for at least 25 minutes. Following the health talk, routine services include weight and height measurement, blood pressure estimation, urinalysis, hemoglobin estimation, and multivitamin supplementation. Following that, patients are requested individually to their physician for medical examination and treatment.

### Study population

The study population consisted of pregnant women of the reproductive age (15-49) attending the antenatal clinic in Plateau Specialist Hospital. Pregnant women who were ill or unable to consent were not included in the study.

### Sampling

The Leslie Kish formula was used to calculate sample size. A precision of 0.05 was used, with an overall client satisfaction prevalence of 81% 19. With a 10% non-response rate, the calculated sample size was 260. However, 238 pregnant women agreed to participate in the study. A convenience sampling technique was used for the study. The sample frame consisted of women who are 15 years above and pregnant. The study unit comprised of pregnant women attending the antenatal clinic. The data collection process took four weeks. Each questionnaire took approximately 15-20 minutes to complete. The interviewers were the principal investigator and three research assistants who had been trained to administer the English-language questionnaire. In cases where the mothers were uneducated, translations into their native language (Hausa) were performed. After the mothers had finished consulting with the doctors, the interview took place.

### Study Variables

The Independent variable is the respondent's perception of the quality of antenatal care while the dependent variable is the level of satisfaction with quality of antenatal care received.

### Study instrument

#### Data collection tool

A semi-structured questionnaire administered by an interviewer was used to collect data. The questionnaire was divided into three sections:

Section A is based on socio-demographic characteristics such as age, ethnicity, and number of children. Section B consisted of question on pregnant women's perception of the quality of antenatal care.

Section C consisted of questions on pregnant women's level of satisfaction with quality of ANC received.

#### Measurement/Operational definition

The Perception of quality of ANC service was measured on a 36-point rating Likert scale of strongly agree, agree, strongly disagree and disagree. Some of the questions were as follows: “attending antenatal care will ensure the safe delivery of my baby”; “No amount of time spent at hospital is wasted”; “People who do not attend antenatal care will have problems in pregnancy” and “antenatal care helps detect pregnancy complications early”. Respondents' perceptions of the quality of ANC were divided into two groups based on the 50th percentile; those who scored 0-18 were considered to have a negative perception of the quality of ANC, while those who scored 19-36 were considered to have a positive perception of the quality of ANC.

The level of satisfaction with the quality of ANC received was measured on a 9-point Likert scale of very satisfied, satisfied, dissatisfied and very dissatisfied. The following questioned were asked: “I will willingly return for follow ups because I am”; “With my experience, I will willingly recommend the facility to a pregnant friend because I am”: “Overall, how can you rate your satisfaction with quality of antenatal care received. Respondents' level of satisfaction with the quality of ANC received was divided into two categories based on the 50th percentile; those who scored 0-4.5 were considered to have a low level of satisfaction with the quality of ANC received, while those who scored 4.6-9 were considered to have a high level of satisfaction with the quality of ANC received.

### Data analysis

The data collected was coded and analysed using the Statistical Package for Scientific Solutions [SPSS] version 23.0 statistical software package. Data was analysed by descriptive and inferential statistics using the information obtained. Association between variables was tested using Pearson's correlation. Finally, the information obtained was summarized and presented in tables for better understanding.

### Ethical considerations

During the study, research ethics issues such as informed consent, anonymity, and confidentiality were carefully addressed. Babcock University Health and Research Ethics Committee (BUHREC) no NHREC/24/01/20138 and Plateau State Specialist Hospital (PSSH) Health and Research Ethics Committee (NHREC/05/01/2010b) approved the study.

## Result

The Response rate was 91.5%.

Socio-demographic distribution of respondent's

The mean age of the respondents was 28.79±6.31. Most (81.9%) of the respondents were married. Forty percent of the respondents belong to the Hausa tribe. Less than half (45.8) of the respondents had tertiary education. Few (28.6%) of the respondents had two children (See, table 1).

**Table 1:** Socio-demographic distribution of respondent's

Characteristics	Category	Frequency	Percentage (%)
Age (in years)		Mean age	
		28.79±6.31	
	19-23	64	26.9
	24-28	51	21.4
	29-33	72	30.3
	34-38	31	13.0
	39-43	14	5.9
	44-48	5	2.1
	49-53	1	0.4
Marital Status			
	Single	20	8.4
	Married	195	81.9
	Divorced	15	6.3
	Separated	8	3.4
Tribe			
	Igbo	48	20.2
	Hausa	95	39.9
	Yoruba	61	25.6
	*Others	34	14.3
Educational level			
	No formal education	10	4.2
	Primary	46	15.1
	Secondary	83	34.9
	Tertiary	109	45.8
Number of children			
	None	42	17.6
	One	40	16.8
	Two	68	28.6
	Three	63	26.5
	Four or more	25	10.5

* *Edo, Idoma, Igala, Berom, Mwagbavul, Tarok*

### Respondents' perception of quality of antenatal care

A little less than half (45.8%) of the respondents strongly agreed that attending antenatal care promote normal pregnancy. More than half (53.4%) of the respondents strongly agreed that attending antenatal care ensure safe delivery. Forty-three percent of the respondents aged that no amount of time spent in the antenatal care clinic is wasted. Fewer than half (45%) of respondents believed that people who did not attend an antenatal clinic would have problems during their pregnancy. In addition, slightly less than half of the respondents (48.7%) strongly agreed that attending an antenatal clinic helps detect pregnancy complications early. Forty-six percent of respondents strongly agreed that receiving antenatal care early in pregnancy treats pregnancy complications. Few respondents (32.2%) strongly disagreed that antenatal care is expensive. Only 16% of respondents strongly agreed that the hospital is a long distance from their home. More than a quarter (41.2%) of respondents strongly agreed that the care they received improved their health and the health of their baby. Forty-two percent of respondents strongly agreed that the information they received assisted them in caring for their pregnancy. Only (13%) of respondents strongly agreed that drugs prescribed during antenatal care were not readily available. Few (18.5%) of respondents strongly agreed that health workers do not answer their pregnancy-related questions. (See, Table 2).

**Table 2:** Respondents' perception of quality of antenatal care

Perception Statement	Strongly agree F (%)	Agree F (%)	Disagree F (%)	Strongly disagree F (%)
Antenatal care ensures that I have a normal pregnancy.	109(45.8)	85(35.7)	37(15.5)	7(2.9)
Attending antenatal care will ensure the safe delivery of my baby.	127(53.4)	90(37.8)	7(7.1)	4(1.7)
No amount of time spent at hospital is wasted.	95(39.9)	104(43.7)	36(15.1)	(1.3)
People who do not attend antenatal care will have problems in pregnancy.	83(34.9)	107(45.0)	39(16.4)	9(3.8)
Antenatal care helps detect pregnancy complications early.	116(48.7)	75(31.5)	40(16.8)	7(2.9)
Antenatal care treats my complications early.	110(46.2)	79(33.2)	31(13.0)	18(7.6)
Antenatal care at the hospital is expensive.	40(16.8)	43(18.1)	78(32.8)	77(32.4)
The distance from my house to the hospital is far.	38(16.0)	58(24.4)	52(21.8)	90(37.8)
My health and that of my unborn baby has improved because of the care given to me at the antenatal clinic.	98(41.2)	79(33.2)	28(11.8)	33(13.9)
The information I receive helps me care for my pregnancy and myself.	100(42.0)	76(31.9)	4(14.3)	28(11.8)
The drugs prescribed to me during antenatal visits are always not available.	31(13.0)	49(20.6)	66(27.7)	92(38.7)
The health workers do not answer questions relating to my pregnancy.	44(18.5)	31(13.0)	53(22.3)	110(46.2)

Furthermore, the respondent's perception of the quality of antenatal care service measured on a 36-point rating scale, showed a mean score of 25.0± 4.61. 6.3% of respondents had a negative perception of the quality of ANC. (See, Table 3)

**Table 3:** Respondents' perception on antenatal care and level of satisfaction

	Respondents in this study; N=238		

	Frequency	Percentage (%)	Mean (SD)
Negative Perception	15	6.3	25.0±4.61
Positive Perception	223	93.7	
Low level of satisfaction	31	13.0	6.50±1.82
High level of satisfaction	207	87.0	

### Respondents level of satisfaction with quality of antenatal care

More than half of the respondents (54.2%) were satisfied with their antenatal care and would return for a follow-up visit. Forty-four percent of respondents reported that they would recommend the facility to a pregnant friend because they were satisfied with the antenatal care they received. Almost half of the respondents (49.2%) were very satisfied with the antenatal care they received (See, Table 4). Also, the respondent's level of satisfaction of antenatal care measured on a 9-point rating scale showed a mean score of 6.50± 1.8. 13% of respondents were dissatisfied with the quality of antenatal care they received. (See, Table 3).

**Table 4:** Respondents level of satisfaction with quality of antenatal care

Statement	Very Satisfied F (%)	Satisfied F (%)	Dissatisfied F (%)	Very Dissatisfied F (%)
I will willingly return for follow ups because I am:	77(32.4)	129(54.2)	23(9.7)	9(3.8)
I will willingly recommend the facility to a pregnant friend because I am:	106(44.5)	85(35.7)	38(16.0)	9(3.8)
Overall, how can you rate your satisfaction with antenatal care received?	86(36.1)	17(49.2)	19(8.0)	16(6.7)

### Relationship between respondents' perception of the quality of antenatal care and level of satisfaction with quality of antenatal care received

The correlation revealed a significant relationship between respondents' perceptions of antenatal care quality and their level of satisfaction with the quality of ANC received. (r =0.433; p< 0.05). (See table 5)

**Table 5:** Relationship between respondents' perception of quality of antenatal care and level of satisfaction with quality of antenatal care received

Variable	Perception of Antenatal care N=238	

	R	p value
**Level of satisfaction**	0.433	0.00

## Discussion

This study examined the perception and level of satisfaction with the quality of antenatal care services among pregnant women at Plateau State Specialist Hospital. Majority of the pregnant women had positive perception of the quality of antenatal care provided at the Plateau State Specialist Hospital. This finding is similar to the findings of a study in Sagamu, Ogun state, where pregnant women reported a positive perception of the quality of ANC services^16^. Also, this finding contradicts the findings of the Ondo and Gambia studies, which found that pregnant women had a negative perception of quality of ANC received 17, 18. Specifically, a significant proportion of clients viewed time spent in the hospital as good. This contradicts findings from Ibadan in Southwest Nigeria^19^. Furthermore, according to Do *et al.*, client satisfaction with the quality of ANC service is an important outcome for health care delivery service 20. According to the findings of this study, respondents were very satisfied with the quality of antenatal care received at Plateau State Specialist Hospital. This is consistent with other studies conducted in Maiduguri, Ibadan, and Kano, where services were deemed adequate. 11, 21, 22. According to some authors, client satisfaction may only reflect low expectations from health care services or a desire to please the interviewer, avoid concerns about provider bias, or express feelings influenced by cultural perceptions 23. As a tertiary hospital, Plateau State Specialist Hospital may provide more specialized care with professionals and consultants. Clients would typically feel more at ease and relieved, which could explain the high level of satisfaction among respondents. However, studies conducted among mothers in Nepal and Ethiopia revealed low levels of satisfaction with the quality of ANC services received 24,25 Disparities in satisfaction scores could be attributed to differences in how services are delivered as well as differences in study populations influencing patient expectations. Cultural differences could also play a role.

### Strength and limitation of the study

Inspite of the findings in this present study, there are few limitations. First, the design was cross-sectional which may not establish a cause-effect relationship. Second, all pregnant women were included irrespective of their previous experience, first time pregnant women may not be able to judge the quality of service effectively. Third, the convenient sampling technique used for selection of the study participants could suggest bias and, also not given everyone equal chance to be selected in the study. Lastly, the study was done in a specialist hospital, this may not be representative of pregnant women who are receiving care at other health facilities both public and private.

## Conclusion

The overall perception of respondents on the quality of ANC has an impact on their level of satisfaction of quality of antenatal care received in Plateau State Specialist Hospital. This study revealed high level of satisfaction with the quality of antenatal care received at the hospital. The main predictors of pregnant women's level of satisfaction with quality of ANC received from this study is their willingness to return for follow up and also recommend the services to their friends. It is recommended that periodic patient satisfaction survey should be established as part of antenatal evaluation to provide feedback for continuous quality improvement.
